# Absence of Thiol-Disulfide Oxidoreductase DsbA Impairs *cbb*_3_-Type Cytochrome *c* Oxidase Biogenesis in *Rhodobacter capsulatus*

**DOI:** 10.3389/fmicb.2017.02576

**Published:** 2017-12-21

**Authors:** Ozlem Onder, Andreia F. Verissimo, Bahia Khalfaoui-Hassani, Annette Peters, Hans-Georg Koch, Fevzi Daldal

**Affiliations:** ^1^Department of Biology, University of Pennsylvania, Philadelphia, PA, United States; ^2^Zentrum für Biochemie und Molekulare Zellforschung (ZBMZ), Institut für Biochemie und Molekularbiologie, Medizinische Fakultät, Albert-Ludwigs-Universität Freiburg, Freiburg, Germany

**Keywords:** disulfide bond formation, copper homeostasis, *cbb*_3_-type cytochrome *c* oxidase biogenesis, *Rhodobacter capsulatus*, respiration, photosynthesis

## Abstract

The thiol-disulfide oxidoreductase DsbA carries out oxidative folding of extra-cytoplasmic proteins by catalyzing the formation of intramolecular disulfide bonds. It has an important role in various cellular functions, including cell division. The purple non-sulfur bacterium *Rhodobacter capsulatus* mutants lacking DsbA show severe temperature-sensitive and medium-dependent respiratory growth defects. In the presence of oxygen, at normal growth temperature (35°C), DsbA^−^ mutants form colonies on minimal medium, but they do not grow on enriched medium where cells elongate and lyse. At lower temperatures (i.e., 25°C), cells lacking DsbA grow normally in both minimum and enriched media, however, they do not produce the *cbb*_3_-type cytochrome *c* oxidase (*cbb*_3_-Cox) on enriched medium. Availability of chemical oxidants (e.g., Cu^2+^ or a mixture of cysteine and cystine) in the medium becomes critical for growth and *cbb*_3_-Cox production in the absence of DsbA. Indeed, addition of Cu^2+^ to the enriched medium suppresses, and conversely, omission of Cu^2+^ from the minimal medium induces, growth and *cbb*_3_-Cox defects. Alleviation of these defects by addition of redox-active chemicals indicates that absence of DsbA perturbs cellular redox homeostasis required for the production of an active *cbb*_3_-Cox, especially in enriched medium where bioavailable Cu^2+^ is scarce. This is the first report describing that DsbA activity is required for full respiratory capability of *R. capsulatus*, and in particular, for proper biogenesis of its *cbb*_3_-Cox. We propose that absence of DsbA, besides impairing the maturation of the *c*-type cytochrome subunits, also affects the incorporation of Cu into the catalytic subunit of *cbb*_3_-Cox. Defective high affinity Cu acquisition pathway, which includes the MFS-type Cu importer CcoA, and lower production of the *c*-type cytochrome subunits lead together to improper assembly and degradation of *cbb*_3_-Cox.

## Introduction

**Figure 1:**
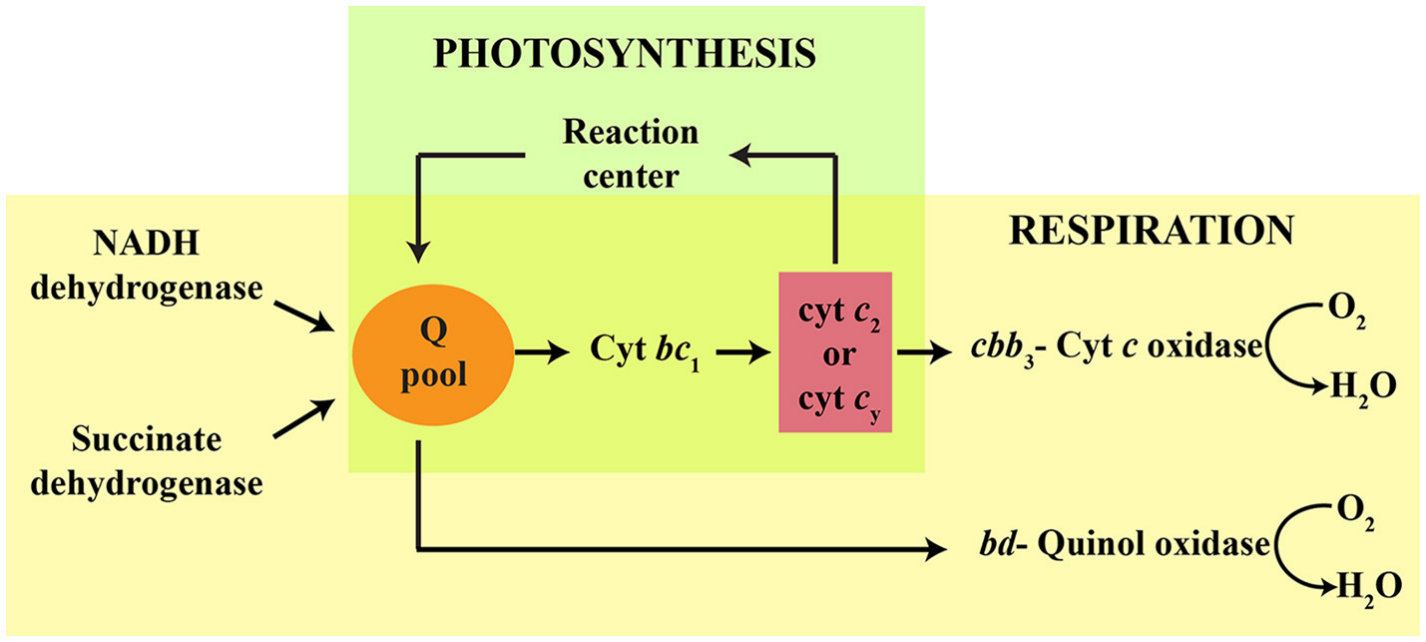
Schematic representation of *R. capsulatus* growth pathways and electron carriers involved in respiration and photosynthesis. The quinol pool (Q pool) is reduced via either the reaction center, or the respiratory NADH and succinate dehydrogenases. During photosynthesis (green), electrons are transferred from the Q pool to cytochrome *bc*_1_ (cyt *bc*_1_). The membrane anchored cyt *c*_y_ and soluble periplasmic cyt *c*_2_ carry electrons from the cyt *bc*_1_ to the reaction center. During aerobic respiration (yellow), the *cbb*_3_-type cyt *c* oxidase is reduced by cyt *bc*_1_ via the electron carrier cyts *c*_2_ or *c*_y_. A cyt *bc*_1_-independent pathway also occurs in *R. capsulatus*, in which the cyt *bd*-quinol oxidase is reduced directly by the Q pool.

The Gram-negative, purple non-sulfur, facultative photosynthetic bacterium *Rhodobacter capsulatus* exhibits versatile growth modes, including anoxygenic photosynthesis (Ps) and aerobic respiration (Res) (Zannoni, 2004). Several *c*-type cytochromes (cyts) and cytochrome (cyt) complexes are key electron transfer components in these energy transduction pathways. Ps growth requires an active photochemical reaction center (RC), an ubihydroquinone: cyt *c* oxidoreductase (cyt *bc*_1_) and an electron carrier (i.e., soluble cyt *c*_2_ or membrane-anchored *c*_y_) (Jenney et al., 1994; Figure 1). Respiratory growth pathways rely on the NADH and succinate dehydrogenases to reduce the quinone (Q) pool that feeds two different terminal oxidases (La Monica and Marrs, 1976; Hochkoeppler et al., 1995). One of these oxidases is a *bd*-type quinol oxidase (*bd*-Qox) that uses hydroquinone (QH_2_) to reduce O_2_ to H_2_O (Giuffrè et al., 2014). The other terminal oxidase is a high O_2_ affinity *cbb*_3_-type cyt *c* oxidase (*cbb*_3_-Cox), which is important for aerobic Res and for the onset of anoxygenic Ps growth (Gray et al., 1994; Hassani et al., 2010). The *cbb*_3_-Cox is a member of a large superfamily of heme-Cu oxygen reductases (García-Horsman et al., 1994; Pereira et al., 2001). It receives electrons from the cyts *c*_2_ or *c*_y_, which are reduced by cyt *bc*_1_ (Hochkoeppler et al., 1995; Daldal et al., 2001), to catalyze O_2_ reduction to H_2_O, while pumping protons across the cytoplasmic membrane (Han et al., 2011; Figure 1). The *cbb*_3_-Cox is composed of four structural subunits (CcoN, CcoO, CcoQ, and CcoP) (Koch et al., 1998), of which CcoO and CcoP are mono- and di-heme *c*-type cyts, respectively (Gray et al., 1994). The catalytic site where O_2_ is converted to H_2_O is located in CcoN, which harbors a low-spin heme *b* and a binuclear center composed of the high-spin heme *b*_3_ and a Cu atom (Cu_B_) (Zufferey et al., 1998; Rauhamäki et al., 2009; Buschmann et al., 2010). CcoQ has no cofactor but is important for the stability of the enzyme (Peters et al., 2008). In wild type *R. capsulatus*, both *cbb*_3_-Cox and *bd*-Qox are active, and either one of them is sufficient to sustain aerobic Res growth (La Monica and Marrs, 1976; Hochkoeppler et al., 1995). Mutants defective for cyt *c* maturation (Ccm) (i.e., covalent addition of heme to the apocyt *c*) (Sanders et al., 2010; Verissimo and Daldal, 2014) do not produce any *c*-type cyt (Sanders et al., 2010; Verissimo and Daldal, 2014), and are unable to grow by Ps, or by Res via the *cbb*_3_-Cox dependent branch. They cannot catalyze the Nadi reaction (α-naphthol + dimethyl-phenylenediamine → indophenol blue + H_2_O) (Nadi^−^ phenotype), which is a visual indicator for the *cbb*_3_-Cox activity (Koch et al., 1998). However, they can still grow by Res via the *bd*-Qox dependent branch, which contains no *c*-type cyt (Aygun-Sunar et al., 2006).

DsbA is a periplasmic thiol-disulfide oxidoreductase that belongs to the thioredoxin family of proteins with a CxxC conserved domain (Shouldice et al., 2011). It facilitates oxidative folding of extra-cytoplasmic proteins by catalyzing the formation of intramolecular disulfide bonds between two reactive Cys residues of its substrates, which include the *c*-type apocyts with their conserved CxxCH heme-binding sites. Reduced DsbA is re-oxidized by its recycling partner DsbB, which then transfers the reducing equivalents to the Q pool, and eventually to the electron transport chain (Inaba and Ito, 2008). In *E. coli*, mutants lacking DsbA are unable to grow under anaerobiosis (Meehan et al., 2017a) and during aerobic growth exhibit a myriad of pleotropic phenotypes, including cell division defects (Meehan et al., 2017b). *R. capsulatus* DsbA^−^ mutants also exhibit complex growth phenotypes (Deshmukh et al., 2003). Under anaerobiosis, they can grow by Ps (Ps^+^), but do not form colonies under aerobiosis on enriched medium at normal growth temperature (35°C). Yet, they revert frequently to bypass this growth defect by acquiring additional mutation(s) (Deshmukh et al., 2003). Comparative proteomic studies showed that the periplasmic protease DegP, which is overproduced in the absence of DsbA, is drastically decreased in these bypass revertants (Onder et al., 2008). These findings indicated that overproduction of DegP is correlated with the growth defect observed in the DsbA^−^ mutants (Spiess et al., 1999; Rizzitello et al., 2001; Onder et al., 2008).

In *R. capsulatus*, and in other organisms, DsbA^−^ mutants are proficient for the Ccm process, but they produce low amounts of *c*-type cyts (Metheringham et al., 1995; Kojima et al., 2005; Turkarslan et al., 2008; Mavridou et al., 2012). In wild type cells, the apocyts *c* are substrates for DsbA. The disulfide bonds formed at their heme-binding sites (CxxCH) are then reduced by a thioredoxin (CcmG)/thiol-disulfide oxidoreductase (CcdA) reductive pathway to allow heme ligation. During this process, reduced apocyts *c* form mixed-disulfide complexes with another thiol-disulfide oxidoreductase (CcmH), which is also likely to be oxidized by DsbA (Verissimo et al., 2017). It is thought that the formation of a disulfide bond at the heme-binding site by DsbA promotes partial folding and confers increased stability to the apocyts *c*, enhancing Ccm efficiency to yield high amounts of *c*-type cyts. In Ccm-deficient mutants, apocyts *c* do not accumulate because they are substrates for the periplasmic protease DegP (Gao and O'Brian, 2007). In the absence of DsbA, the apocyts *c* remain reduced and the CcmG/CcdA-catalyzed reductive pathway becomes unnecessary for Ccm (Deshmukh et al., 2003; Turkarslan et al., 2008). However, as the absence of DsbA leads to the overproduction of the periplasmic protease DegP, a fraction of the apocyts *c* are most likely degraded before they are able to ligate heme, resulting in ~50% decrease in the steady-state amounts of the *c*-type cyts (Onder et al., 2008; Turkarslan et al., 2008).

Proper assembly of *cbb*_3_-Cox does not only require a functional Ccm system but is also dependent on Cu availability. How Cu is acquired from the environment and incorporated into the active site of *cbb*_3_-Cox during its biogenesis is not well understood. In *R. capsulatus* a high affinity Cu uptake pathway has been described. It involves various transporters, such as CcoA (Ekici et al., 2014) and CcoI (Koch et al., 2000), and chaperones, including PccA (Trasnea et al., 2016) and SenC (Swem et al., 2005; Lohmeyer et al., 2012). Mutants lacking any of these components have very small amounts of *cbb*_3_-Cox, and in most cases, this defect can be palliated by addition of Cu^2+^ to the growth medium (Ekici et al., 2012a). Moreover, a low affinity Cu uptake pathway was also observed, but its components remain unknown (Ekici et al., 2014).

In this study, we analyzed the properties of *R. capsulatus* DsbA^−^ mutants. We found that they form filamentous and osmosensitive cells at 35°C under Res growth conditions on enriched medium, where bioavailable Cu is limited. In contrast, these mutants grow normally at 25°C, but they produce very low levels of *cbb*_3_-Cox. However, upon supplementation of the growth media with redox-active chemicals, they can grow normally and produce active *cbb*_3_-Cox. Our results showed that overproduction of the Cu importer CcoA partially restores the *cbb*_3_-Cox defect, suggesting defective Cu incorporation into this enzyme in the absence of DsbA. This is the first description of DsbA being required for respiratory capabilities of *R. capsulatus*, and in particular, for efficient *cbb*_3_-Cox biogenesis under conditions where bioavailable Cu^2+^ is scarce.

## Materials and methods

### Bacterial strains, plasmids, and growth conditions

The bacterial strains and plasmids used in this work are described in Table 1. *E. coli* strains were grown aerobically at 37°C in LB medium, supplemented with ampicillin (Amp), kanamycin (Kan), spectinomycin (Spe) or tetracycline (Tet) as needed, at final concentrations of 100, 50, 50, or 12.5 μg per ml, respectively. *R. capsulatus* strains were grown chemoheterotrophically (i.e., by Res in the dark) at either 35°C or 25°C, in Sistrom's minimal medium A (MedA) (Sistrom, 1960) or in enriched medium (MPYE, mineral-peptone-yeast extract), supplemented with 10, 10 and 2.5 μg per ml of Kan, Spe or Tet, respectively (Darrouzet et al., 2002). As needed, up to 20 μM CuSO_4_ was added to MPYE to obtain “MPYE+Cu,” and the 1.5 μM CuSO4, which is normally present in MedA, was omitted from MedA to obtain “MedA-Cu” media (Ekici et al., 2012b). Alternatively, mixtures containing different concentrations of cysteine and cystine were used as redox supplements (Turkarslan et al., 2008). For induction of CcoA expression in the DsbA^−^ mutant carrying a plasmid containing wild type *ccoA* (BK69/MD20, Table 1), 1% L-arabinose was added to the medium.

**Table 1:** Bacterial strains and plasmids used in this study.

**Strain/plasmid**	**Description**	**Relevant phenotype**	**Source/References**
**STRAINS**			
***E. coli***			
HB101	F^−^Δ(*gpt-proA*)62 *leuB*6 *supE*44 *ara-14 galK*2 *lacY1* Δ(*mcrC-mrr*) *rpsL*20 *xyl-*5 *mtl-*1 *recA*13	Str^R^, ${\text{r}}_{\text{B}}^{-}$ ${\text{m}}_{\text{B}}^{-}$	Sambrook and Russell, 2001
XL1-Blue	F'::Tn*10 proA^+^B^+^ lacI*^q^ Δ(*lacZ*)M15/*recA1 endA1 gyrA96* (Nal^R^) *thi hsdR17* (${\text{r}}_{\text{K}}^{-}$ ${\text{m}}_{\text{K}}^{+}$) *supE44 relA1 lac*		Stratagene
***R. capsulatus***			
MT1131^a^	*crtD121* Rif ^R^	Wild type, Res^+^ Nadi^+^ Ps^+^^b^	Scolnik et al., 1980
SB1003	Wild type	Wild type, Res^+^ Nadi^+^ Ps^+^	Yen and Marrs, 1976
Y262		GTA overproducer	Yen et al., 1979
MD20	Δ(*dsbA*::*kan)*	Res^+^ Nadi^+^ Ps^+^ on Med A^c^ Res^Ts^ Nadi^−^ Ps^+^ on MPYE^c^	Deshmukh et al., 2003
ST20	Δ(*dsbA*::*spe)*	similar to MD20 but Spe^R^	This work
SE8	Δ(*ccoA::spe*)	Res^+^ Nadi^−^ Ps^+^ on MPYE Res^+^ Nadi^+^ Ps^+^ on MedA	Ekici et al., 2012a
GK32	Δ(*ccoNO::kan*)	Res^+^ Nadi^−^ Ps^+^	Koch et al., 1998
KZ1	Δ(*Q_*ox*_*::spe)	Res^+^ Nadi^+^ Ps^+^	Zhang and Daldal
LS01	Δ(*senC*) derivative of SB1003	Res^+^ Nadi^−^ Ps^+^ on MPYE Res^+^ Nadi^+^ Ps^+^ on MedA	Swem et al., 2005
DS1	Δ(*dsbA::kan*) Δ(*Q_*ox*_::spe*) derivative of KZ1	Like MD20, except Ps^+^ Res^−^ on all media	This work
OZL4	Δ(*dsbA*::*spe)* Δ(*ccoNO::kan*) derivative of GK32	Like MD20, except Res^+^ Nadi^−^ on all media	This work
**PLASMIDS**			
pRK2013	*tra*^+^ (RK2)	Kan^R^, helper	Ditta et al., 1985
pRK415	Broad host-range vector	Tet^R^	Keen et al., 1988
pBluescript	Cloning vector, pBluescript II KS (+)	Amp^R^	Stratagene
pHP45ΩSpe	Ω*spe* in pHP45 vector	Spe^R^, Amp^R^	Prentki and Krisch, 1984
pTC4-1K	4.2 kb *Xba*I-*Kpn*I insert carrying *dsbA*::*kan* allele cloned into the corresponding sites of pRK415	Kan^R^, Tet^R^	Deshmukh et al., 2003
pKZ2	Δ(*Qox::spec*) allele in pRK415	Spe^R^, Tet^R^	Zhang and Daldal
p4AiK32	Δ(*ccoNO::kan*) in pRK415	Kan^R^, Tet^R^	Koch et al., 1998
pOX15	Wild type ccoNOQP in pRK404	Tet^R^	Koch et al., 1998
pDsbA^wt^	Wild type *dsbA* carried by pRK415	Tet^R^	Deshmukh et al., 2003
pBK69	Wild type *ccoA* carried by pRK415-pBAD	Amp^R^, Tet^R^	Khalfaoui-Hassani et al., 2016a
pSenCFlagC	Wild type *senC* with a C-ter Flag epitope carried by Prkm	Kan^R^	Onder et al., 2008

a *R. capsulatus strain MT1131 was originally isolated as a “green” (i.e., carrying crtD121 mutation) derivative of R. capsulatus wild type SB1003 (Scolnik et al., 1980). All mutants are derivative of MT1131 unless mentioned otherwise*.

b *Ps and Res refer to photosynthetic and respiratory growth ability, respectively. Nadi indicates Cox-dependent ability to catalyze α-naphthol to indophenol blue. Res^Ts^ refers to the ability to grow at 25°C but not at 35°C*.

c *MedA and MPYE refer to minimal and enriched growth media, respectively*.

The *cbb*_3_-Cox activity on colonies was visualized using the Nadi staining reaction (formation of indophenol blue) by overlaying them with a mixture containing 1:1 (v/v) of 35 mM α-naphthol and 30 mM *N, N, N',N'*-dimethyl-*p*-phenylene diamine (Marrs et al., 1972). Colonies with *cbb*_3_-Cox activity exhibited dark blue staining (Nadi^+^) within 30 s to 1 min, while those lacking it showed no staining (Nadi^−^) up to 15 min (Aygun-Sunar et al., 2006).

To obtain photomicrographs of *R. capsulatus* cells, overnight cultures were sub-cultured on enriched medium at 25°C for ~6 h under Res conditions, and then switched to 35°C for 4 h. A 5 μl culture sample was deposited on a microscope slide, and cells were immobilized under a cover slip with a drop of 1% agarose. Slides were examined using an Olympus IX81 microscope at a magnification of 1,000× (10× objective and 100× ocular) and representative field images were acquired with a SensiCam QE camera (Cooke Corporation, Romulus, MI) and IPLab version 4.0 software (Scanalytics, Fairfax, VA).

### Molecular genetic techniques

Molecular genetic techniques were performed according to standard protocols (Sambrook and Russell, 2001). Conjugal transfer of plasmids from *E. coli* to *R. capsulatus*, and interposon mutagenesis via the gene transfer agent (GTA) (Yen et al., 1979) using the Spe^R^ gene cassette from pHP45Ω (Prentki and Krisch, 1984), were carried out as described earlier (Daldal et al., 1986). The DsbA^−^ *cbb*_3_-Cox^−^ and DsbA^−^ *bd*-Qox^−^ double mutants were obtained by GTA crosses using as donors the *ccoNO::spe* or *cydAB::spe* alleles carried by the plasmids pAiK32 (Koch et al., 1998) or pKZ2 (Zhang and Daldal, unpublished), respectively (Table 1). Strain MD20 (Δ*dsbA::kan*) (Deshmukh et al., 2003) was used as a recipient, selecting for antibiotic resistance under growth permissive conditions (e.g., MPYE+Cu plates at 25°C). The double mutants thus obtained were tested for their temperature sensitive Res growth (Res^Ts^) and Cu^2+^-suppressible phenotypes on MPYE at 35°C. Appropriate merodiploids were constructed by introducing the plasmids pDsbA, pSenC and pBK69 (CcoA) carrying wild type alleles of *dsbA, senC* and *ccoA*, respectively into the DsbA^−^ and DsbA^−^ SenC^−^mutants (Table 1) using triparental crosses as described earlier (Valkova-Valchanova et al., 1998).

### Biochemical techniques

As required, *R. capsulatus* total cell extracts were prepared using cells collected from freshly grown plates of enriched medium to avoid any undesired reversion event. Cells were resuspended in 25 mM Tris pH 7.5, 150 mM NaCl, 2 mM 4-(2-Aminoethyl) benzenesulfonyl fluoride hydrochloride (AEBSF), 1% N-dodecyl-β-D-maltoside (DDM), 0.2 mg/mL lysozyme and 0.05 mg/mL DNase lysis buffer and incubated at room temperature for 20 min. Clarified extracts were obtained after centrifugation at 15,000 × *g* for 15 min. Chromatophore (i.e., intracytoplasmic) membranes were prepared from cells grown in liquid enriched medium under Res conditions for ~24 h, and stored at −80°C until use. Cells were disrupted using a French pressure cell in 50 mM MOPS pH 7.0, 10 mM EDTA, 1 mM phenylmethylsulfonylfluoride (PMSF), 0.1 mM ε-aminocaproic acid buffer, as described earlier (Sanders et al., 2008; Verissimo et al., 2011). After 2 h centrifugation at 138,000 × g and 4°C, membrane pellets were resuspended in different buffers as needed. *R. capsulatus* chromatophore membranes were resuspended to a final concentration of 1–5 mg/ml in 10 mM Tris-HCl pH 7.0, 120 mM KCl buffer and dispersed with a protein: detergent ratio of 1:1 (w/w) of DDM. Protein concentrations were determined according to the Bradford (Bio-Rad) or the BCA (Sigma-Fisher) methods, using bovine serum albumin as a standard. Protein samples were resolved using 12.5% Tris-Glycine (Laemmli, 1970) or 16.5% Tris-Tricine (Schägger and von Jagow, 1987) SDS-PAGE or blue native (BN)-PAGE (Schägger and von Jagow, 1991). The CcoN subunit of *cbb*_3_-Cox was identified by incubating immunoblots with rabbit polyclonal antibodies against *R. capsulatus* CcoN (Koch et al., 2000), following Tris-Glycine SDS-PAGE and electrotransfer onto Immobilon-PVDF membranes (Millipore, MA). Horseradish peroxidase-conjugated anti-rabbit IgG (GE Healthcare) was used as a secondary antibody, and detection was done using the SuperSignal West Pico Chemiluminescent Substrate (Thermo Scientific, Inc.). The presence of *c*-type cyts in the protein samples was detected via the endogenous peroxidase activity of their covalently attached hemes, using 3,3′,5,5-tetramethylbenzidine (TMBZ) following Tris-Tricine SDS-PAGE (Thomas et al., 1976). Semi-quantitative estimation of the amount of *c*-type cyts and CcoN subunits of *cbb*_3_-Cox was done using the ImageJ software.

### Enzymatic assays

The *cbb*_3_-Cox and cyt *bc*_1_ activities were measured using a Hitachi U-3210 spectrophotometer by monitoring oxidation, or reduction, of horse heart cyt *c* (Sigma, MO), respectively, in a stirred cuvette at 25°C. Horse heart cyt *c* (1 mM) was reduced with sodium dithionite, and excess of reductant removed using a PD-10 desalting column (GE Healthcare). Determination of *cbb*_3_-Cox activity was done using cells grown either in solid enriched medium at 25°C under Res condition, or in liquid enriched medium for ~24 h at 25°C under Res conditions. The assays were initiated by adding to the assay buffer containing 50 μM of reduced cyt *c* either 100–200 μg of *R. capsulatus* total protein extracts (prepared from freshly grown cells in solid medium) or 1–10 μg of DDM-dispersed chromatophore membrane proteins (prepared from cells grown in liquid medium and stored at −80°C until breakage using a French pressure cell). The decrease in absorbance at 550 nm due to oxidation of cyt *c*, was recorded for 2 min. The specificity of the assay for *cbb*_3_-Cox activity was confirmed by addition of 0.1 mM KCN to the assay mixture, which immediately stopped cyt *c* oxidation (Gray et al., 1994). Note that the *cbb*_3_-Cox is the only Cox enzyme present in *R. capsulatus* membranes (Hochkoeppler et al., 1995). Cyt *bc*_1_ activity was measured using 40 μM decylbenzoquinol (DBH_2_) as an electron donor and 50 μM cyt *c* as an electron acceptor as described elsewhere (Atta-Asafo-Adjei and Daldal, 1991). The reaction was initiated by addition of 50–250 μg of DDM-dispersed chromatophore membrane proteins to the assays mixture and the increase in absorbance at 550 nm due to cyt *c* reduction was monitored for 1 min. The portion of the initial rate that was inhibited by addition of 150 μM famoxadone was used as the specific cyt *bc*_1_ activity.

O_2_ consumption rates exhibited by *R. capsulatus* membranes were determined polarographically at 25°C using a Clark-type oxygen electrode (Yellow Springs Instruments, Co) and appropriate electron donors and inhibitors. All assays were carried out using 2 ml of 50 mM MOPS, 5 mM MgCl_2_, pH 7.2 assay buffer, containing 50–300 μg of DDM-dispersed membrane proteins from cells grown in liquid MPYE enriched medium for ~24 h at 25°C under Res conditions (Valkova-Valchanova et al., 1998). Depending on the segment of the respiratory chain to be assayed, the O_2_ consumption reaction was initiated by addition of 1 mM NADH (for NADH dehydrogenase), 200 μM DBH_2_ (for *bd*-Qox) or 250 μM N,N,N',N'-tetramethyl-*p*-phenylenediamine (TMPD) together with 10 mM sodium ascorbate (for *cbb*_3_-Cox) (Hochkoeppler et al., 1995). For *bd*-Qox activity measurements, chromatophore membranes were previously incubated with 150 μM famoxadone and 100 μM KCN to inhibit the cyt *bc*_1_ and *cbb*_3_-Cox activities, respectively. In all assays, the respiratory chain independent O_2_ consumption activity was determined by using 2 mM KCN, which completely inhibits both *cbb*_3_-Cox and *bd*-Qox dependent O_2_ reductase activities, and subtracted from the respiratory rates observed. Enzymatic activities were expressed in μmoles of O_2_ reduced per min, per mg of total protein, considering that air-saturated buffer at 25°C contains 0.237 mM O_2_. The temperature sensitivity of a chosen respiratory enzyme was tested by incubating chromatophore membranes for 2 h at 35°C prior to O_2_ consumption assays.

The β-galactosidase activities of strains carrying the *ccoN::lacZ* were visualized qualitatively using enriched medium plates containing 40 μg/ml of the chromogenic indicator 5-bromo-4-chloro-3-indolyl-β-D-galactopyranoside (X-Gal). The β-galactosidase activities were also assayed at 420 nm using liquid cultures grown in enriched medium at 25°C, with or without addition of 5 μM CuSO4, as described earlier (Khalfaoui-Hassani et al., 2016a). Briefly, bacteria were centrifuged and resuspended in 1 ml of Z-buffer (60 mM Na_2_HPO_4_ pH 7.0, 40 mM NaH_2_PO_4_, 10 mM KCl, 1 mM MgSO_4_ and 50 mM β-mercaptoethanol), and their OD_600_ nm was recorded. The cells were permeabilized using 100 μl of chloroform and 50 μl of 0.1% SDS. The reaction was started by adding 0.2 ml of *o*-nitrophenyl-β-galactoside (ONPG; 4 mg/ml), incubated at 30°C for 20 min, and stopped by adding 0.5 ml of 1 M Na_2_CO_3_. The absorbance at 420 nm resulting from the pigments of *R. capsulatus* cells was recorded before the addition of ONPG, and subtracted from the final absorbance read at 420 nm at the end of incubation. The β-galactosidase activity was expressed as μmoles of ONPG hydrolyzed per minute per OD_600_ of cells according to ΔOD_420_ × 10^6^/4,860/min/OD_600_ of cells, as done earlier (Khalfaoui-Hassani et al., 2016a).

### Chemicals

All chemicals were of highest purity and HPLC spectral grades, and purchased from commercial sources.

## Results

### *R. capsulatus* DsbA^−^ mutants are temperature sensitive for respiratory growth

**Figure 2:**
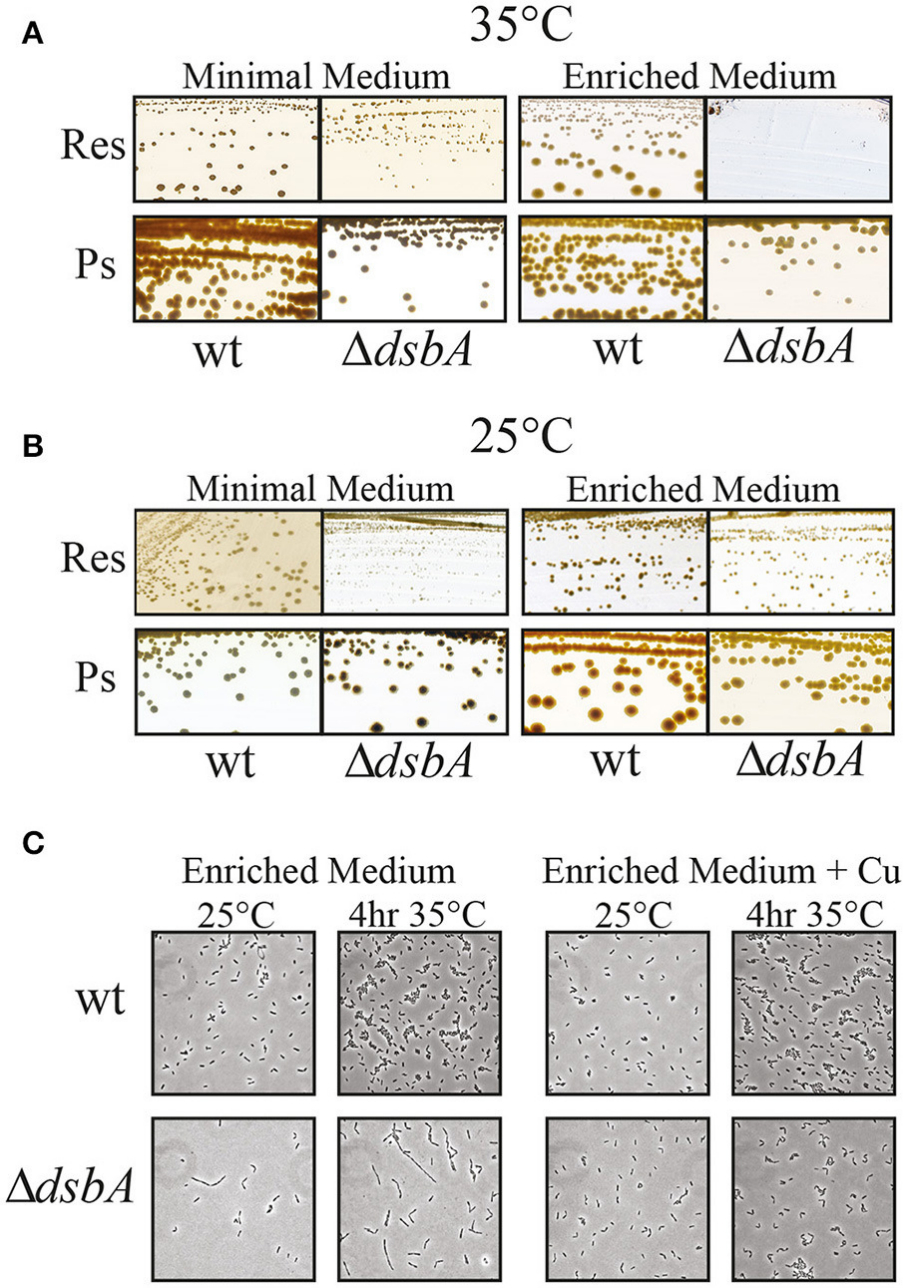
The Res growth of DsbA^−^ mutants is temperature-sensitive. *R. capsulatus* wild type strain and Δ*dsbA* mutant grown in minimal (MedA) and in enriched medium (MPYE) at 35°C **(A)** and 25°C **(B)**. The respiratory growth (Res) of Δ*dsbA* is defective when the cells are grown at 35°C on enriched medium. DsbA^−^ mutant is still able to grow on minimal medium and under Ps growth conditions, albeit at lower rate than wild type. **(C)** Microscopic examination of wild type and Δ*dsbA* mutant cells with or without addition of 5 μM of Cu^2+^ (+Cu) in the enriched medium. Cell division is severely affected in the Δ*dsbA* mutant, producing filaments that are longer than the wild type cells. This growth phenotype is suppressed by the addition of Cu^2+^ into the growth medium.

Previously, we had observed that *R. capsulatus* mutants lacking DsbA were able to grow via Ps, albeit at a slower rate, on both enriched and minimal growth media, but could grow by Res only on minimal, and not on enriched medium, at normal temperature (35°C) (Figure 2A; Deshmukh et al., 2003; Turkarslan et al., 2008). Moreover, the DsbA^−^ mutants reverted readily on enriched medium at 35°C to regain Res growth ability. Proteomic analyses showed that in the absence of DsbA the protease DegP was overproduced, and that the revertants contained mutations that lowered DegP activity (Onder et al., 2008). DegP is usually less abundant and acts as a chaperone at lower temperatures (Spiess et al., 1999). Considering this unusual functional switch, we tested the growth ability of *R. capsulatus* DsbA^−^ mutant at 25°C in enriched medium. We found that a DsbA^−^ mutant formed colonies at this lower temperature, indicating that the absence of DsbA rendered *R. capsulatus* cells temperature sensitive for Res growth on enriched medium (Res^Ts^) (Figure 2B, right side and Table 2). Upon microscopic examination of liquid cultures, we also observed that the DsbA^−^ mutants exhibited defective cell division. Cells growing by Res in enriched medium at 25°C stopped dividing when shifted to 35°C, yielding elongated cells that eventually lysed (Figure 2C, left side).

**Table 2:** Growth phenotypes of *R. capsulatus* DsbA^−^ single and double mutants in minimal and enriched medium, with or without supplementation with Cu^2+^, grown at 25 or 35°C.

**^a^Strains**	**35°C**				**25°C**			
	**Minimal Medium**	**^a^Minimal Medium −Cu**	**Enriched Medium**	**Enriched Medium +Cu**	**Minimal Medium**	**Minimal Medium −Cu**	**Enriched Medium**	**^a^Enriched Medium +Cu**
Wild type	Res^+^ ^b^Nadi^+^	Res^+^ Nadi^+^	Res^+^ Nadi^+^	Res^+^ Nadi^+^	Res^+^ Nadi^+^	Res^+^ Nadi^+^	Res^+^ Nadi^+^	Res^+^ Nadi^+^
Δ*dsbA*	Res^+^ Nadi^+^	^c^NG ^d^na	NG Na	Res^+^ Nadi^+^	Res^+^ Nadi^+^	Res^+^ Nadi^−^	Res^+^ Nadi^−^	Res^+^ Nadi^+^
Δ(*dsbA)* Δ(*cbb*_3_-Cox)	Res^+^ Nadi^−^	NG na	NG Na	Res^+^ Nadi^−^	Res^+^ Nadi^−^	Res^+^ Nadi^−^	Res^+^ Nadi^−^	Res^+^ Nadi^−^
Δ(*dsbA*) Δ(*bd*-Qox)	Res^+^ Nadi^+^	NG na	NG Na	Res^+^ Nadi^+^	Res^+^ Nadi^+^	NG na	NG na	Res^+^ Nadi^+^

a *All mutants are proficient for Ps growth in all media and temperatures although “pre-reduced” media may provide better Ps growth. Res^+^ or Res^−^ refers to the presence or absence of respiratory growth, respectively. Minimal medium-Cu and Enriched medium+Cu indicate omission and addition of Cu^2+^, respectively*.

b *Nadi^+^ or Nadi^−^ refers to the presence or absence of cbb_3_-Cox activity of colonies as revealed by the Nadi staining, respectively*.

c *NG, no growth*.

d *na, not applicable*.

### *R. capsulatus* respiratory chain is not temperature sensitive in the absence of DsbA

**Figure 3:**
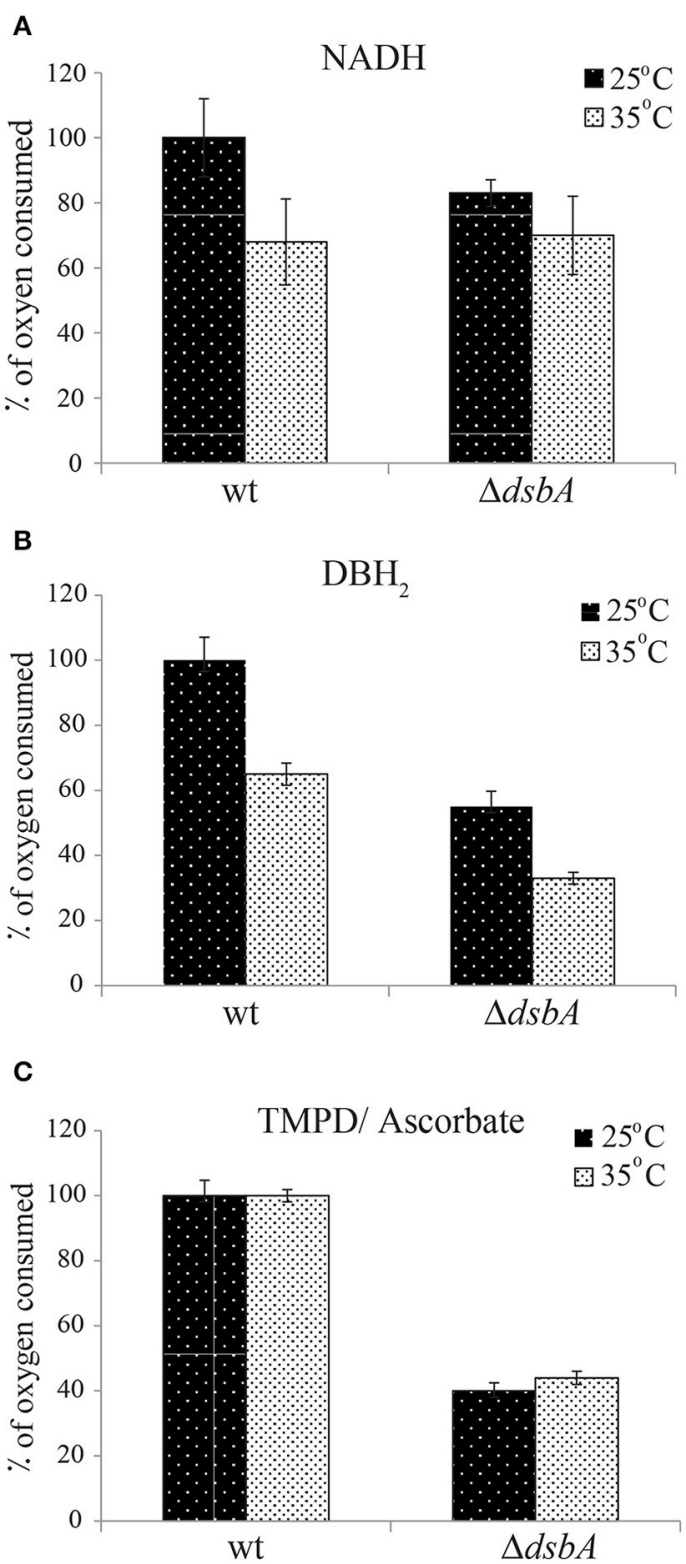
Enzymatic activities of the respiratory complexes are not temperature-sensitive in the absence of DsbA. The rates of oxygen consumption were determined polarographically at 25°C using a Clark-type oxygen electrode, in DDM-solubilized chromatophore membranes from cells grown in liquid enriched medium at 25°C under Res conditions. All assays were carried out using 2 ml of 50 mM MOPS, 5 mM MgCl_2_, pH 7.2 assay buffer, containing 50–300 μg of DDM-dispersed membrane proteins [protein: detergent (w/w) of 1:1] from wild type and Δ*dsbA* strains. Depending on the segment of the respiratory chain to be assayed, the O_2_ consumption reaction was initiated by addition of 1 mM NADH (for NADH dehydrogenase) **(A)**, 200 μM DBH_2_ (for *bd*-Qox) **(B)** or 250 μM N,N,N',N'-tetramethyl-*p*-phenylenediamine (TMPD) together with 10 mM sodium ascorbate (for *cbb*_3_-Cox) **(C)**. The same measurements at 25°C were also repeated after incubation of the membranes at 35°C for 2 h (gray bars) to assess the temperature response of the activities. The rates were determined as μmoles of O_2_ reduced/min/mg total protein, and are shown as % of oxygen consumed taking as 100% the rate of the wild type strain. Average values of at least 3 independent measurements are shown. Error bars represent the range of values (maximum and minimum) obtained for each condition.

In order to probe whether the Res^Ts^ phenotype of *R. capsulatus* DsbA^−^ mutants originated from temperature sensitivity of their respiratory chain component(s), we polarographically monitored the O_2_ consumption rates of wild type and Δ*dsbA* strains. Cells were grown under Res conditions in liquid enriched medium (MPYE) at 25°C for 24 h and stored at −80°C. Chromatophore membranes were isolated and solubilized with DDM, and the rate of O_2_ consumption was assayed at 25°C (Materials and Methods). The measurements were repeated using solubilized membranes that were incubated for 2 h at 35°C prior to the assays. NADH oxidase, DBH_2_ oxidase (*bd*-Qox, in the presence of a cyt *bc*_1_ inhibitor) and TMPD/Ascorbate-dependent O_2_ reductase (*cbb*_3_-Cox) activities were measured using appropriate substrates and inhibitors. The data showed that in such cells the respiratory capabilities of the DsbA^−^ mutant were variable but always lower than those of the wild type strain, and the *bd*-Qox and *cbb*_3_-Cox activities were affected to a greater extent than the NADH dehydrogenase activity (Figure 3). When membranes were incubated at 35°C and then assayed at 25°C, the NADH dehydrogenase and *bd*-Qox activities decreased similarly in both strains (Figures 3A,B). However, the *cbb*_3_-Cox activity remained unchanged both in wild type and DsbA^−^ strains, indicating that this enzyme was not temperature sensitive (Figure 3C). Thus, although the absence of DsbA decreased the overall activities of the respiratory enzymes, it did not render them more temperature sensitive than their wild type counterparts. Hence, the Res^Ts^ phenotype of the DsbA^−^ mutant was not correlated with the temperature sensitivity of the respiratory chain components.

### Respiratory growth at 25°C of a DsbA^−^ mutant is mediated by *bd*-Qox enzyme

We further examined the Res growth pathways of the DsbA^−^ mutant at 25°C in enriched medium, to assess whether growth was sustained either by the *cbb*_3_-Cox, or *bd*-Qox, or both of these terminal oxidases (Figure 1). *R. capsulatus* double mutants lacking both DsbA and either of the *cbb*_3_-Cox or the *bd*-Qox were constructed under the permissive growth conditions (Materials and Methods). As expected, neither strain could grow on enriched medium at 35°C, confirming their DsbA^−^ phenotypes. However, the DsbA^−^ *cbb*_3_-Cox^−^ double mutant was able to grow at 25°C on both enriched and minimal media, whereas the DsbA^−^ *bd*-Qox^−^ double mutant could do so only on minimal, but not on enriched medium (Table 2). Thus, the *bd*-Qox activity sustained the Res growth on enriched medium at 25°C, indicating that in the absence of DsbA, the *cbb*_3_-Cox activity became defective even under growth permissive conditions. Together with the temperature insensitivity of the *bd*-Qox activity described above, this finding implied that the Res growth defect observed in the DsbA^−^ mutant was not due to a defective *bd*-Qox activity.

### Redox active chemicals palliate growth and *cbb*_3_-Cox defects of DsbA^−^ mutants

**Figure 4:**
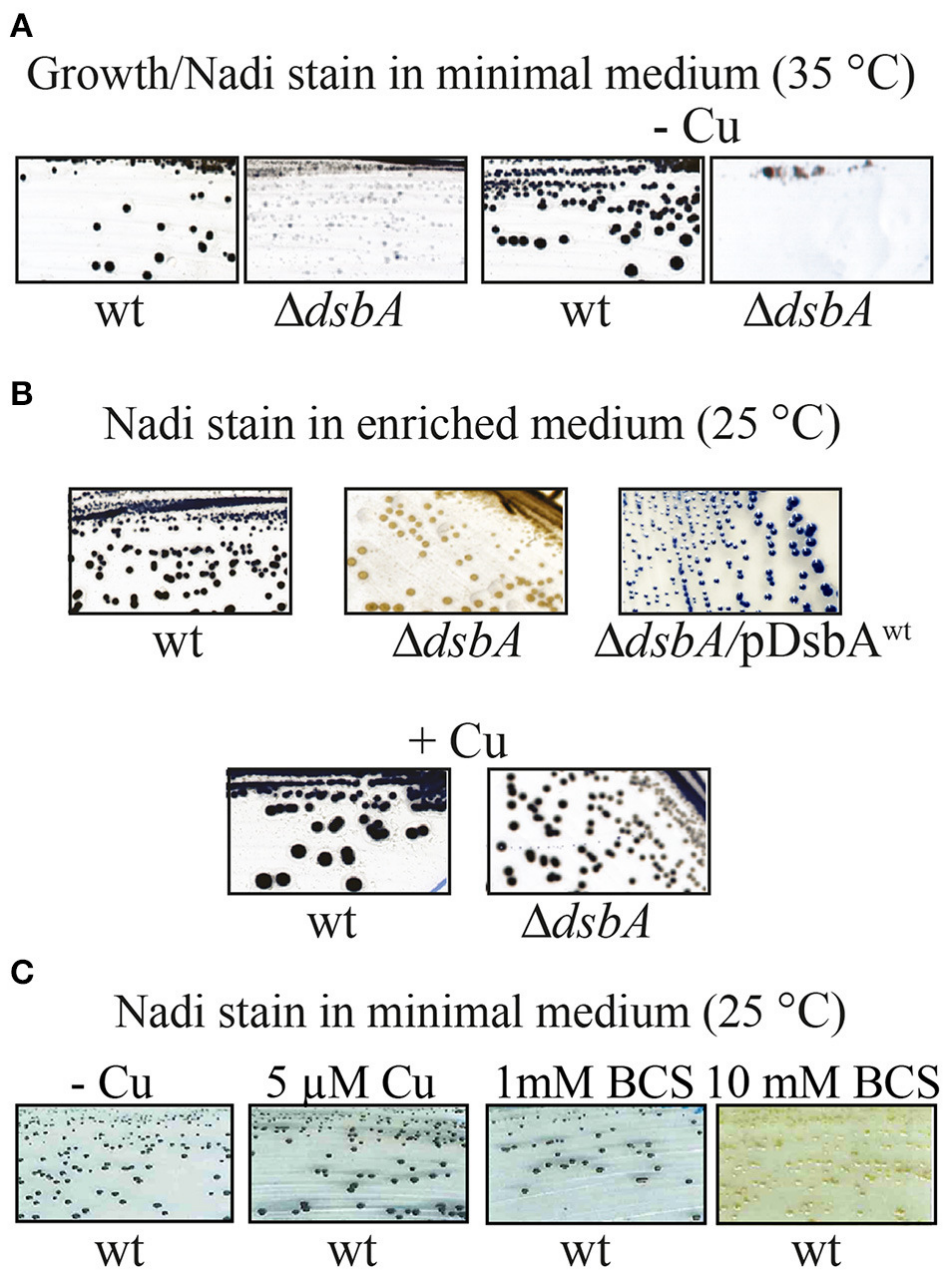
The NADI phenotype of a DsbA^−^ mutant in *R. capsulatus*. **(A)** Wild type and Δ*dsbA* mutant cells grown on minimal medium (MedA) at 35°C. The Δ*dsbA* grows at lower rate than wild type, and when Cu^2+^ is omitted from the minimal medium (-Cu) the Δ*dsbA* mutant does not grow. Both wild type and Δ*dsbA* mutant cells become blue after Nadi staining. **(B)** Nadi staining of wild type and Δ*dsbA* mutant in enriched medium (MPYE) at 25°C. The Δ*dsbA* mutant does not show any NADI staining and this phenotype is suppressed by addition of Cu^2+^. Complementation of the Δ*dsbA* mutant with wild type *dsbA* restores Nadi staining, indicating restored production of *cbb*_3_-Cox. **(C)** Nadi staining of wild type cells grown on minimal medium without Cu^2+^ (-Cu), with addition of 5 μM of Cu^2+^, or with addition of 1 mM or 10 mM of the Cu chelator bathocuproine disulfonate (BCS). A wild type strain becomes Nadi^−^ (no blue stain), like the Δ*dsbA* or Δ*cbb*_3_-Cox mutants, when Cu^2+^ in the medium is chelated by BCS.

We observed frequently that the *R. capsulatus* DsbA^−^ mutants that were grown on minimal medium showed partial growth at 35°C when transferred to enriched medium. However, this growth ability was not sustained for subsequent cultures in the enriched medium, suggesting that carryover of a chemical(s) beneficial to growth, from the minimal to the enriched medium might be responsible for these observations. Systematic supplementation of the enriched medium with the constituents of the minimal medium, as well as omission of these constituents from the minimal medium, identified Cu^2+^ as the culprit. Indeed, addition of 5 μM Cu^2+^ to the enriched medium allowed normal cell division (Figure 2C, right side, bottom row), restoring growth and *cbb*_3_-Cox defects at 35°C. Similarly, omission of Cu^2+^ from the minimal medium (normally containing 1.5 μM Cu^2+^) rendered the DsbA^−^ mutants Res^Ts^ at 35°C (Figure 4A), and addition of Cu^2+^ to enriched medium rendered it Nadi^+^ at 25°C (Figure 4B). Interestingly, addition of 10 mM of the Cu chelator bathocuproine disulfonate (BCS) to the minimal medium also rendered wild type cells Nadi^−^ without affecting their growth ability at 25°C, thus mimicking the phenotype of a DsbA^−^ mutant (Figure 4C). These findings suggested that in the absence of DsbA, Cu^2+^ incorporation into *cbb*_3_-Cox might be defective.

Suppression of growth and *cbb*_3_-Cox defects was specific to Cu^2+^ ions only, as no other component of the minimal medium, including several other transition metal ions (e.g., Fe^3+^, Ni^2+^, and Mn^2+^) could remedy these defects (data not shown). Titration of the Cu^2+^ amount added to the enriched medium (between 0 and 500 μM) or the minimal medium (0–10 μM) at 25 and 35°C showed that higher amounts of Cu^2+^ were required in enriched medium compared with the minimal medium to suppress the DsbA^−^ mutant growth and *cbb*_3_-Cox defects (Table 3). These observations were in accordance with the restricted bio-availability of Cu^2+^ in enriched medium (MPYE) containing yeast extract (rich in metal chelators), as previously reported (Park et al., 1998). Interestingly, while addition of lower amounts of Cu^2+^ in both media was sufficient to rescue growth at 35°C, higher amounts were needed to restore the *cbb*_3_-Cox activity (i.e., Nadi^+^) (Table 3). Similarly, when enriched medium was supplemented with a mixture of cysteine/cystine, lower concentrations allowed cell growth but higher concentrations were needed to rescue the Nadi phenotype (Table 4). We also noted that, like the *E. coli* DsbA^−^ mutants (Hiniker et al., 2005), the *R. capsulatus* DsbA^−^ mutants were more sensitive to Cu^2+^ than other strains (Table 3).

**Table 3:** Respiratory growth and Nadi phenotypes of various *R. capsulatus* strains grown in minimal or enriched media at 25 or 35°C supplemented with increasing Cu^2+^ concentrations.

**Strains^a^**	**35°C/Enriched medium**						**25°C/Enriched medium**					
**Cu^2+^ (μM)^a^**	**0**	**1**	**10**	**20**	**100**	**500**	**0**	**1**	**10**	**20**	**100**	**500**
WT	^b^Res^+^ ^c^Nadi^+^	Res^+^ Nadi^+^	Res^+^ Nadi^+^	Res^+^ Nadi^+^	Res^+^ Nadi^+^	Res^+^ Nadi^+^	Res^+^ Nadi^+^	Res^+^ Nadi^+^	Res^+^ Nadi^+^	Res^+^ Nadi^+^	Res^+^ Nadi^+^	Res^+^ Nadi^+^
*ΔdsbA*	^b^NG ^d^na	NG Na	Res^+^ Nadi^+^	Res^+^ Nadi^+^	NG Na	NG Na	Res^+^ Nadi^−^	Res^+^ Nadi^−^	Res^+^ Nadi^+^	Res^+^ Nadi^+^	NG Na	NG Na
*ΔsenC*	Res^+^ Nadi^−^	Res^+^ ^e^Nadi^S/?^	Res^+^ Nadi^+^	Res^+^ Nadi^+^	Res^+^ Nadi^+^	NG Na	Res^+^ Nadi^−^	Res^+^ Nadi^S/?^	Res^+^ Nadi^+^	Res^+^ Nadi^+^	Res^+^ Nadi^+^	NG Na
	**35**°**C/Minimum medium**						**25**°**C/Minimum medium**					
**Cu**^2+^ **(**μ**M)**	**0**	**1**	**1.5**	**2**	**5**	**10**	**0**	**1**	**1.5**	**2**	**5**	**10**
WT	Res^+^ Nadi^+^	Res^+^ Nadi^+^	Res^+^ Nadi^+^	Res^+^ Nadi^+^	Res^+^ Nadi^+^	Res^+^ Nadi^+^	Res^+^ Nadi^+^	Res^+^ Nadi^+^	Res^+^ Nadi^+^	Res^+^ Nadi^+^	Res^+^ Nadi^+^	Res^+^ Nadi^+^
*ΔdsbA*	NG Na	Res^S^ Nadi^s/?^	Res^+^ Nadi^+^	Res^+^ Nadi^+^	Res^+^ Nadi^+^	Res^+^ Nadi^+^	NG Na	Res^S^ Nadi^s/?^	Res^+^ Nadi^+^	Res^+^ Nadi^+^	Res^+^ Nadi^+^	Res^+^ Nadi^+^
*ΔsenC*	Res^+^ Nadi^+^	Res^+^ Nadi^+^	Res^+^ Nadi^+^	Res^+^ Nadi^+^	Res^+^ Nadi^+^	Res^+^ Nadi^+^	Res^+^ Nadi^+^	Res^+^ Nadi^+^	Res^+^ Nadi^+^	Res^+^ Nadi^+^	Res^+^ Nadi^+^	Res^+^ Nadi^+^

a *All strains are Ps^+^ in all media and temperatures. All media are supplemented with indicated concentrations of CuSO_4_*.

b *Res^+^ and NG refers to normal or no respiratory growth, respectively*.

c *Nadi^+^ or Nadi^−^ refers to the presence or absence of cbb_3_-Cox activity of colonies as revealed by Nadi staining*.

d *na, not applicable*.

e *Nadi^s/?^ indicates slow/unreliable Nadi staining due to slow Res growth*.

**Table 4:** Res growth and Nadi phenotypes of various *R. capsulatus* strains grown in minimal or enriched media at 35°C supplemented with increasing concentrations (mM) of a mixture of cysteine/cystine.

**^a^Strains**	**35**°**C/Minimal medium**				**35**°**C/Enriched medium**			
**Cysteine/Cystine mixture (mM)**	**0**	**0.33/0.165**	**0.5/0.25**	**1.0/0.5**	**0**	**0.33/0.165**	**0.5/0.25**	**1.0/0.5**
WT	^b^Res^+^ ^c^Nadi^+^	Res^+^ Nadi^+^	Res^+^ Nadi^+^	Res^+^ Nadi^+^	Res^+^ Nadi^+^	Res^+^ Nadi^+^	Res^+^ Nadi^+^	Res^+^ Nadi^+^
*ΔdsbA*	Res^+^ Nadi^+^	Res^+^ Nadi^+^	Res^+^ Nadi^+^	Res^+^ Nadi^+^	^b^NG ^d^na	Res^+^ Nadi^−^	Res^+^ Nadi^+^	Res^+^ Nadi^+^

a *All strains are Ps^+^ in all media*.

b *Res^+^ and NG refers to normal or no respiratory growth, respectively*.

c *Nadi^+^ or Nadi^−^ refers to the presence or absence of cbb_3_-Cox activity of colonies as revealed by Nadi staining, respectively*.

d *na, not applicable*.

When tested immediately after growth in agar containing (i.e., solid) enriched medium (MPYE) at 25°C without Cu^2+^ supplementation, the DsbA^−^ mutants showed very low *cbb*_3_-Cox activity (i.e., Nadi^−^) (Figure 2B, Table 5), unlike the cells grown in liquid medium (Figure 3 and Table 5). However, upon storage of plates at room temperature and exposed to air, or after growth in liquid medium they regained partially *cbb*_3_-Cox activity (Figure 3 and Table 5). The molecular basis of this difference was not investigated, but it might be caused by “phenotypic and/or genotypic” reversion of the DsbA^−^ mutants in liquid cultures (Onder et al., 2008). The amount of oxygen present in shaken liquid cultures grown under Res conditions might partially alleviate the oxidative defect due to the absence of DsbA. Conversely, the presence of agar on solid media might exacerbate this defect. Indeed, complementation of the DsbA^−^ mutant with wild type DsbA (pDsbA^wt^), or supplementation of the enriched medium with Cu^2+^ (Figure 4B) or cysteine/cystine mixture (data not shown) acting as chemical oxidants allowed growth and mitigated the *cbb*_3_-Cox defect at 35°C (i.e., Nadi^+^). The DsbA^−^ mutants grown at 25°C in enriched medium in the presence of ~0.66 mM cysteine and 0.33 mM cystine or 5–10 μM Cu^2+^ contained higher *cbb*_3_-Cox activity than those grown without any supplement, which relied uniquely on oxygen to compensate for the absence of DsbA (Table 5). We also noted that the DsbA^−^ mutants had lower cyt *bc*_1_ activity under Res growth conditions, despite their ability to grow by Ps under anaerobiosis, and this defect was also palliated by addition of chemical oxidants (Table 5). Thus, the DsbA^−^ mutants exhibited a general redox imbalance that impaired multiple cellular functions, including its respiratory activities and cell division.

**Table 5:** Cyt *c*: oxygen oxidoreductase activity of *cbb*_3_-Cox and DBH_2_: cyt *c* oxidoreductase activity of cyt *bc*_1_ of *R. capsulatus* wild type, *cbb*_3_-Cox^−^ and DsbA^−^ strains.

**Strains**	^a^**Tested immediately after growth in solid enriched medium (MPYE)**		^b^**Tested after storage and cell growth in liquid enriched medium (MPYE)**			
	***cbb*_3_-Cox activity**	**% wt activity**	***cbb*_3_-Cox activity**	**% wt activity**	**cyt *bc*_1_ activity**	**% wt activity**
Wild type	78 ± 12	100	0.95 ± 0.10	100	1.30 ± 0.10	100
*Δcbb*_3_*-Cox*	0.41 ± 0.2	0.5	^c^nd	^c^na	nd	na
*ΔdsbA*	4.80 ± 0.6	6.0	0.55 ± 0.10	58	0.25 ± 0.05	20
*ΔdsbA* + CC	nd	na	0.85 ± 0.08	90	1.20 ± 0.10	92
*ΔdsbA* + Cu^2+^	nd	na	0.95 ± 0.10	100	nd	na

a *Strains were grown by respiration at 25°C in solid enriched medium (MPYE), colonies were collected from plates, lysed and cell extracts were assayed immediately. Activities were determined as nmoles of cyt c ox/min/mg of total proteins*.

b *Alternatively, strains were grown in liquid enriched medium (MPYE) with or without supplementation of chemical oxidants [10 μM Cu^2+^ or a mixture of 0.66 mM cysteine and 0.33 mM cystine (CC)], and cells were collected and stored at −80°C until chromatophore membrane preparation and activity measurements. Activities were determined as μmoles of cyt c oxidized or reduced/min/mg of membrane proteins. In both cases, activity measurements were done using DDM-solubilized samples (Materials and Methods)*.

c *nd, not determined and na, not applicable*.

### Mutants lacking DsbA have lower amounts of the *cbb*_3_-Cox subunits

Given that Cu^2+^ supplementation of a DsbA^−^ mutant increased its *cbb*_3_-Cox activity, and that chelation by BCS eliminated this activity in a wild type strain, we inquired whether Cu^2+^ affected the expression of the *ccoNOQP* operon, encoding the structural genes of *cbb*_3_-Cox. A transcriptional-translational *ccoN::lacZ* fusion, containing the promoter region of *ccoNOQP* and the first eight amino acids of CcoN (Koch et al., 2000) was introduced into the wild type and DsbA^−^ strains. At 25°C, these strains formed colonies of comparable blue colors on 5-bromo-4-chloro-3-indoyl β-D-galactopyranoside (X-gal) containing enriched medium plates (data not shown). Similarly, in liquid cultures they produced comparable amounts of β-galactosidase activities in the absence or presence of 5 μM Cu^2+^ (Table 6). Thus, neither the addition of Cu^2+^, nor the absence of DsbA significantly affected *ccoNOQP* expression, suggesting that the lack of *cbb*_3_-Cox activity was not due to impaired transcription or initiation of translation of its CcoN subunit.

**Table 6:** β-galactosidase activity of *R. capsulatus* wild type and DsbA^−^ strains determined in cell cultures, grown by respiration at 25°C in enriched medium (MPYE) in the presence or absence of 5 μM of Cu^2+^.

**Strains**	**25**°**C/Enriched medium**	
	**No Cu^2+^ added**	**5 μM of Cu^2+^**
WT	28 ± 0.3	28 ± 2.8
*ΔdsbA*	26 ± 1.3	24 ± 2.2

*Activities are expressed as μmoles of ONPG hydrolyzed per minute per OD_600_ of cells*.

**Figure 5:**
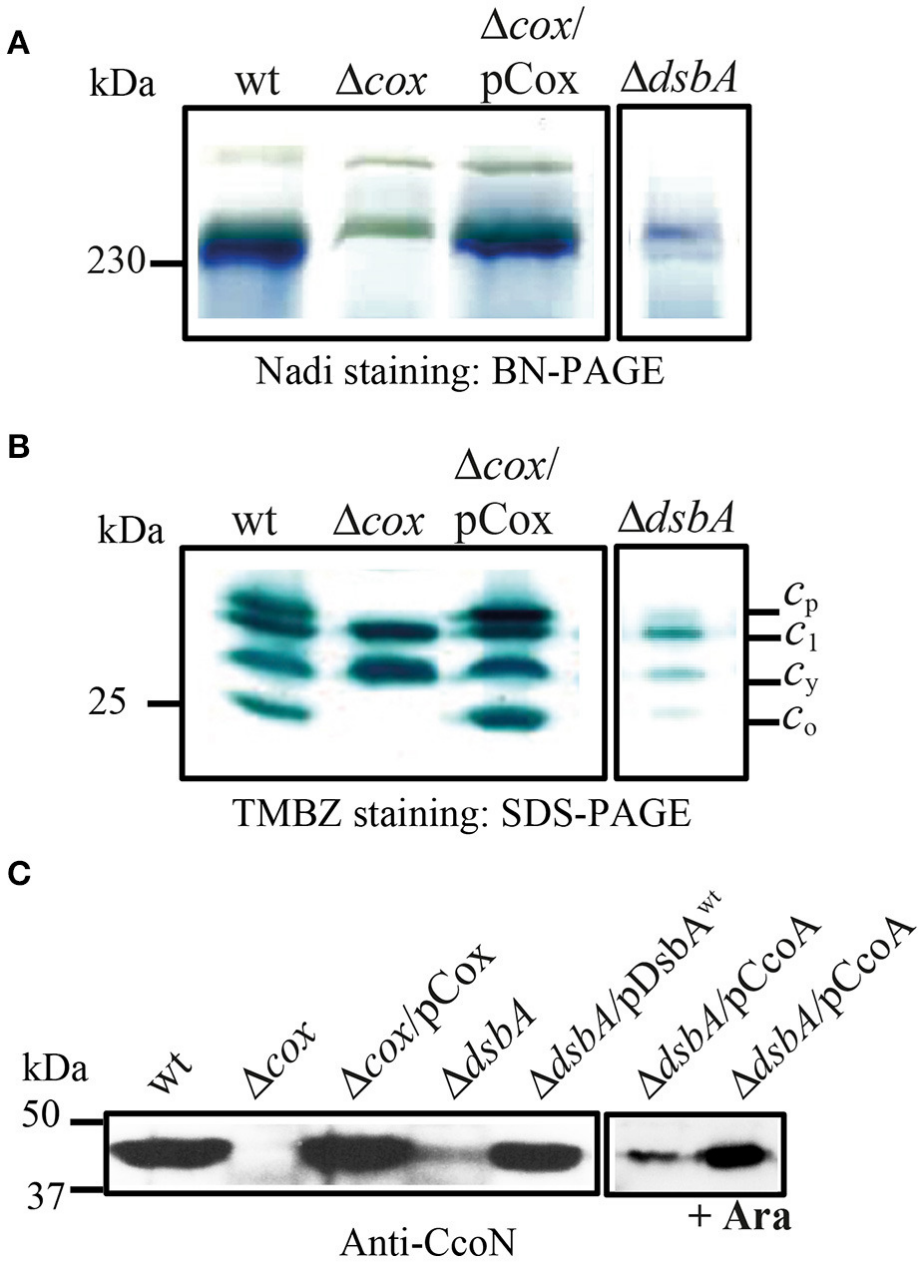
DsbA is required for *cbb*_3_-Cox activity and maturation/assembly of its subunits. **(A)** Detection of active *cbb*_3_-Cox by BN-PAGE stained with NADI in chromatophore membranes prepared from the wild type (wt), Δ*cbb*_3_-Cox (Δ*cox*), Δ*cbb*_3_-Cox overproducing *cbb*_3_-Cox (Δ*cox*/pCox) and Δ*dsbA*. The active 230 kDa band corresponding to *cbb*_3_-Cox complex is present in the wild type and Δ*cox*/pCox strains. In the absence of DsbA, activity of the *cbb*_3_-Cox is severely affected. **(B)** Chromatophore membranes were separated by SDS-PAGE and stained by TMBZ for detection of the *c*-type cyts. The wild type and Δ*cox*/pCox showed cyt *c*_1_ of cyt *bc*_1_ complex, the electron carrier *c*_y_, and the cyts *c*_p_ and *c*_o_ subunits of *cbb*_3_-Cox, which are absent in Δ*cbb*_3_-Cox. The levels of all *c*-type cyts *c* are decreased in Δ*dsbA* due to its effect on the Ccm process, and the cyts *c*_p_ and *c*_o_ subunits of *cbb*_3_-Cox were present at ~10 and 8% of the wild type levels, respectively. **(C)** Immunoblot analysis of chromatophore membranes separated by SDS-PAGE using polyclonal antibodies against the CcoN subunit of *cbb*_3_-Cox. The amount of CcoN is severely reduced in the Δ*dsbA* mutant to ~15% of wild type levels, but it increases by roughly three folds when CcoA is overproduced upon induction with L-arabinose.

In order to further investigate why the production of an active *cbb*_3_-Cox was defective in the absence of DsbA, chromatophore membranes of cells grown at 25°C in enriched medium (MPYE) were analyzed by native BN-PAGE and by denaturing SDS-PAGE. Activity staining of native gels using the Nadi reaction for *cbb*_3_-Cox revealed an intense band of ~230 kDa, corresponding to *cbb*_3_-Cox activity in the wild type and in the *cbb*_3_-Cox^−^ mutant complemented with a plasmid carrying wild type *ccoNOQP* genes (Δ*cox*/pCox) (Kulajta et al., 2006), but not in the *cbb*_3_-Cox^−^ (Δ*cox*) or DsbA^−^ mutants (Figure 5A). Similarly, denaturing gels followed by TMBZ staining showed in wild type membranes four *c*-type cyts, *c*_p_, *c*_1_, *c*_y_, and *c*_o_ (Figure 5B). In the absence of DsbA, the amounts of the cyts *c*_1_, *c*_y_, *c*_p_, and *c*_o_ decreased drastically in freshly grown cells to 40, 18, 10, and 8% of the wild type levels, respectively, as indicated by semi-quantitative analysis using ImageJ software. Lastly, immunoblot analysis using *R. capsulatus* anti-CcoN polyclonal antibodies showed that the steady-state level of the catalytic subunit CcoN (with a Cu_B_ center and no *c*-type cyts) was also very low in a DsbA^−^ mutant (about 15% of the wild type) (Figure 5C), similar to the other subunits of *cbb*_3_-Cox. Upon complementation of the DsbA^−^ mutant with a plasmid carrying wild type DsbA (Δ*dsbA*/pDsbA), the levels of CcoN increased to wild type levels (Figure 5C). While we anticipated decreased amounts of the *c*-type cyts in the absence of DsbA, due to its role during Ccm (Deshmukh et al., 2003; Turkarslan et al., 2008), finding a decrease in the Cu containing CcoN, which is not a *c*-type cyt, was unexpected. Considering that CcoN alone is highly stable in the absence of its *c*-type cyt partners (Ekici et al., 2013), provided that CcoQ is present (Peters et al., 2008), these observations further suggested that *R. capsulatus* DsbA^−^ mutants were defective in Cu^2+^ incorporation into *cbb*_3_-Cox. The very low levels of this enzyme were then a consequence of the overproduction of DegP, as indicated by the nature of the suppressor mutations that lowered this protease activity (Onder et al., 2008).

### DsbA^−^ and several *cbb*_3_-Cox biogenesis mutants exhibit similar phenotypes

We noticed that the *cbb*_3_-Cox related properties of the DsbA^−^ mutants were reminiscent of some of the *cbb*_3_-Cox biogenesis mutants such as those lacking the Cu importer CcoA (Ekici et al., 2012b) or the Cu chaperones SenC and PccA (Lohmeyer et al., 2012; Trasnea et al., 2016; Table 3). Earlier studies indicated that CcoA, PccA, and SenC are required for Cu incorporation into *cbb*_3_-Cox at low Cu availability, and mutants lacking these components had small amounts of *cbb*_3_-Cox, which could be restored by supplementation of the growth medium with Cu^2+^ (Ekici et al., 2012a). The Cu chaperone PccA has no cysteine residues, but SenC which is homologous to the mitochondrial Sco proteins involved in Cox biogenesis, contains two cysteines required for Cu binding (Thompson et al., 2012; Trasnea et al., 2016). Similarly, CcoA also has several cysteine residues and at least one is critical for its function (unpublished data). These resemblances led us to probe for possible functional link(s) between DsbA and CcoA or SenC, provided that these proteins could be substrates of DsbA. We constructed the pDsbA^+^/SenC^−^ and pSenC^+^/DsbA^−^ as well as the pDsbA^+^/CcoA^−^ and pCcoA^+^/DsbA^−^ (pBK69/MD20) merodiploids that carried multiple copies of wild type *dsbA, senC* or *ccoA* alleles (Table 1). Indeed, no functional complementation was seen between DsbA and SenC or DsbA and CcoA with respect to the Res^Ts^ phenotype, consistent with the Res^Ts^ defect being unrelated to *cbb*_3_-Cox biogenesis. Interestingly, although no complementation was observed between SenC and DsbA (pSenC/MD20 or pDsbA/LS01), in respect to the Nadi^−^ phenotype at 25°C in enriched medium, a partial complementation was detected when CcoA was overproduced in the DsbA^−^ mutant upon L-Ara induction of wild type *ccoA* carried by a plasmid (pBK69/MD20) (Khalfaoui-Hassani et al., 2016a) (i.e., the Nadi phenotype became Nadi^S^) (Table 1). Considering that CcoA is important for Cu incorporation into the CcoN subunit of *cbb*_3_-Cox, immunoblot analyses were carried out using CcoN antibodies. The data showed that, the overproduction of CcoA as a result of arabinose induction in the DsbA^−^ mutant increased about three times the amount of CcoN in the membranes (as determined using ImageJ software) consistent with the enhanced Nadi staining of this merodiploid strain (Figure 5C). This finding implied that the *cbb*_3_-Cox defect was at least partly due to a shortage of active CcoA and compromised Cu incorporation into its catalytic site. Thus, DsbA has a hitherto unknown role during *cbb*_3_-Cox biogenesis, especially under restricted Cu availability conditions.

## Discussion

**Figure 6:**
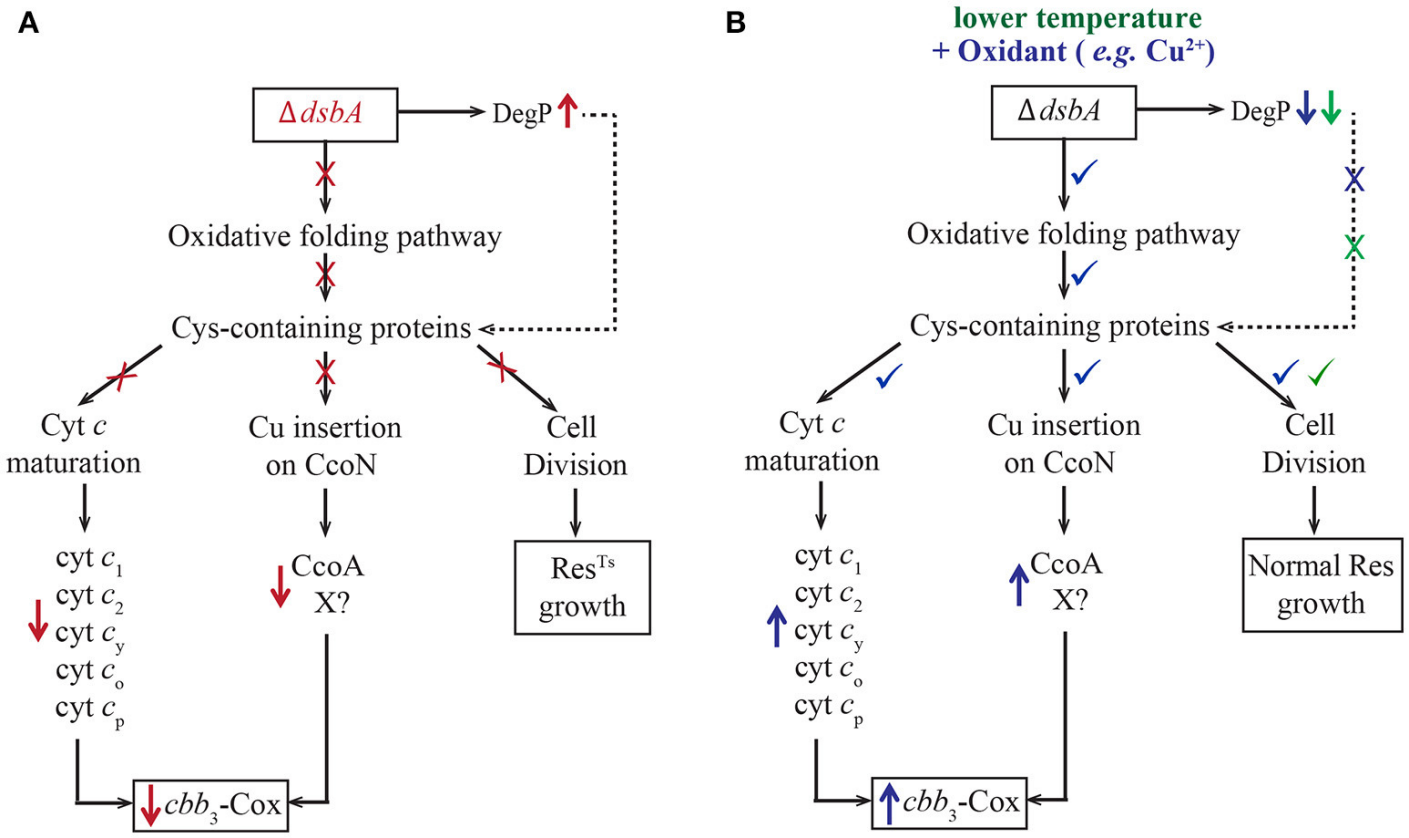
Absence of DsbA has multiple effects on *R. capsulatus* respiratory growth. **(A)** In the absence of DsbA (shown in red), the oxidative folding pathway is defective, causing improper folding of many Cys-containing proteins. These proteins are important for many cellular functions ranging from cyt *c* maturation (Ccm) (left), Cu incorporation into CcoN (middle) and cell division (right) that become defective (marked with ×). In addition, in the absence of DsbA, the periplasmic protease DegP is overproduced (marked with ↑), and degrades rapidly misfolded DsbA substrates. The impairment of cell division leads to the Res^TS^ growth phenotype (right). The defect on Ccm results in lower steady-state amounts of the *c*-type cyts (*c*_1_, *c*_2_, *c*_y_, *c*_o_, *c*_p_) and their complexes, including the cyt *bc*_1_ and *cbb*_3_-Cox (left) (marked with ↓). Other Cys-containing proteins, such as CcoA and hypothetical components (X) involved in Cu incorporation into the catalytic subunit CcoN, also become targets of DegP, further decreasing *cbb*_3_-Cox production (marked with ↓) (middle). **(B)** Lowering the growth temperature (shown in green) decreases the protease activity of DegP (marked with ↓), improving cell division (marked with ✓), but not *cbb*_3_-Cox biogenesis. However, addition of a chemical oxidant, such as Cu^2+^ or a mixture of cysteine/cysteine (shown in blue), repairs the redox imbalance due to the absence of DsbA, decreases the levels of DegP (marked with ↓) and improves folding of Cys-containing proteins (marked with ✓). This in turn allows growth at normal temperature (35°C) (right), efficient Ccm (left) and enhances *cbb*_3_-Cox biogenesis (marked with ✓) (middle). In addition, use of high concentrations of Cu^2+^ also activates the low affinity Cu uptake pathway, which facilitates Cu insertion into the CcoN subunit (not shown).

Previously, we have shown that *R. capsulatus* DsbA^−^ mutants do not grow in enriched medium (MPYE) by Res although they are proficient for Ps growth (Deshmukh et al., 2003). We investigated the properties of such mutants, and found that their growth defects were linked to cellular redox imbalance and resulting defective oxidative protein folding, which led to decreased *c*-type cyt maturation and impaired *cbb*_3_-Cox biogenesis (Figure 6A, in red). Improperly folded proteins are targets to the periplasmic protease DegP, which is overproduced in the DsbA^−^ mutant. At 35°C, cell division in a DsbA^−^ mutant stopped, yielding elongated cells that eventually lysed. The observation that *R. capsulatus* cells could grow at 35°C in minimal medium, but not in enriched medium, led us to find that exposure to oxygen and to redox-active chemicals (e.g., cysteine/cystine mixture or Cu^2+^ ions) restored the redox imbalance due to the absence of DsbA (Figure 6B, in blue). Supplementation of the growth medium with Cu^2+^ enhanced disulfide bond formation in DsbA^−^ mutants. However, since the chemically catalyzed process is less specific than the DsbA-catalyzed disulfide bond formation, cells relied on the disulfide bond isomerase DsbC for survival (Hiniker et al., 2005), and became more Cu^2+^ sensitive. Additionally, lowering the growth temperature (from 35 to 25°C) also alleviated the growth, but not the *cbb*_3_-Cox, defect of the *R. capsulatus* DsbA^−^ mutant (Figure 6B, in green). The ability to grow at low temperature indicated that decreased DegP activity and oxygen mediated basal disulfide bond formation were sufficient for Res growth via the *bd*-Qox pathway. Accordingly, addition of small amounts of redox chemicals was sufficient to rescue the growth defect, but higher amounts were needed to observe wild type-like *cbb*_3_-Cox biogenesis.

Remarkably, the growth defects associated with a DsbA^−^ mutant of *R. capsulatus* (a soil/aquatic organism) were not identical to those observed with an analogous *E. coli* (an enteric organism) mutant. The DsbA^−^ mutants of both species exhibited cell division defects under aerobic Res conditions where residual protein disulfide bond formation presumably relied on oxygen (Meehan et al., 2017b). Recently, it was shown that the *E. coli* cell division protein FtsN, which has a structural disulfide bond, is rapidly degraded in the absence of DsbA, leading to cell filamentation (Meehan et al., 2017b). FtsN is present only in a few bacterial species, and is absent in *R. capsulatus* (https://biocyc.org), although it is plausible that a functional analog might exist in this organism. Furthermore, an *E. coli* DsbA^−^ mutant is unable to grow under anaerobic conditions, possibly due to defective outer membrane biosynthesis (Meehan et al., 2017b) and deficient cyt *c* maturation (Metheringham et al., 1995). In contrast, the *R. capsulatus* DsbA^−^ mutant is proficient for anaerobic growth in the presence of light (i.e., photosynthesis) (Deshmukh et al., 2003). Indeed, the two species have different anaerobic growth modes, while *E. coli* relies mainly on glycolysis, *R. capsulatus* performs anoxygenic photosynthesis (Hunter et al., 2009).

Perhaps the most salient finding of this work is the decrease of the respiratory capacity observed in a mutant lacking DsbA (Figure 6A). In particular, the cyt *bc*_1_ and *cbb*_3_-Cox activities were much lower in these cells that grew by Res via the *bd*-Qox enzyme. It is known that DsbA is not essential for Ccm, but its absence decreases the steady-state levels of *c*-type cyts by ~50% (Turkarslan et al., 2008). Occurrence of a thio-redox loop (oxidation of an apocyt *c* by DsbA followed by its reduction by CcmG) might protect the unfolded apocyt *c* against degradation via the holdase role of CcmG (Turkarslan et al., 2008). Our recent work showed that CcmG interacts with CcmH and other Ccm components involved in heme ligation. In a DsbA^−^ mutant, CcmH that is responsible for the stereo-specificity of heme ligation may not be rapidly oxidized. This would compromise the formation of correct thioether bonds, leading to degradation of non-native cyts *c* (Verissimo et al., 2017). In addition to the lower efficiency of the Ccm process, we also note that two of the cyt *bc*_1_ subunits, the FeS protein (Valkova-Valchanova et al., 1998) and the cyt *c*_1_ (Gray et al., 1992) contain disulfide bonds that are essential for their stability and function. Thus, the lower cyt *bc*_1_ activity found in a DsbA^−^ mutant could be due in part to incomplete formation of these structural disulfide bonds. Although two of the cyt *cbb*_3_-Cox subunits are *c*-type cyts, they do not contain any structural disulfide bond (Koch et al., 2000). Thus, the decreased Ccm efficiency was not sufficient to rationalize why *cbb*_3_-Cox was quasi-absent in a DsbA^−^ mutant. Moreover, it was unclear why the CcoN subunit of *cbb*_3_-Cox, which is matured independently of the Ccm process, and stable in the absence of the cyts *c*_o_ and *c*_p_ subunits (Ekici et al., 2013), was barely detectable in the absence of DsbA. In addition, we did not observe in native gels the presence of a fully-assembled, but inactive cyt *cbb*_3_-Cox, in contrast to what was observed in *cbb*_3_-Cox biogenesis mutants lacking CcoS (Koch et al., 2000; Kulajta et al., 2006). These observations, combined with the unaffected expression of the *ccoNOQP* cluster and the remedial of *cbb*_3_-Cox defect by decreased DegP activity (Onder et al., 2008) implied that the *cbb*_3_-Cox subunits were absent in a DsbA^−^ mutant due to their rapid degradation (Figure 6A). Our earlier comparative proteomics study showed that *R. capsulatus* cells lacking DsbA contained lower amounts of several multi-cysteine containing periplasmic proteins compared to wild type, suggesting that without the formation of their critical disulfide bonds, these proteins remain unfolded, leading to their elimination via increased DegP activity (Onder et al., 2008). While some of these DsbA-dependent proteins are important for essential cellular functions such as osmo-protection, outer membrane synthesis or lipid biosynthesis (Onder et al., 2008), conceivably, other yet to be identified components (X) might be involved in *cbb*_3_-Cox biogenesis (Figure 6A).

The presence of small quantities of CcoN in membranes is a common characteristic of *cbb*_3_-Cox biogenesis mutants that are defective for incorporation of Cu into c*bb*_3_-Cox at low Cu^2+^ availability (Ekici et al., 2012a; Khalfaoui-Hassani et al., 2016b). Cells lacking the MFS-type Cu importer CcoA and the periplasmic Cu chaperones SenC and PccA (Koch et al., 2000; Lohmeyer et al., 2012; Ekici et al., 2014; Trasnea et al., 2016) contain very low levels of *cbb*_3_-Cox subunits and activity, and like the DsbA^−^ mutants (Hiniker et al., 2005), are phenotypically rescued by addition of Cu^2+^. Aside from PccA, both SenC (Lohmeyer et al., 2012) and CcoA contain Cys residues required for their activity (unpublished data), possibly forming structurally or functionally important disulfide bonds. In the absence of DsbA, the active forms of these, and probably other, proteins may be quasi-absent, leading to impaired Cu incorporation into *cbb*_3_-Cox especially at low Cu^2+^ bioavailability (Figure 6A). Consistent with this possibility is the fact that a wild type strain of *R. capsulatus* also becomes impaired in *cbb*_3_-Cox biogenesis when grown in the presence of BCS, which restricts Cu availability.

How Cu is incorporated into the CcoN subunit of *cbb*_3_-Cox is not well understood. A high affinity Cu uptake pathway involving at least CcoA and SenC, and a CcoA-independent low affinity Cu uptake pathway have been described (Ekici et al., 2012a). Our data suggests that, in the absence of DsbA, the naturally bioavailable Cu^2+^ present in enriched medium is not efficiently incorporated into the *cbb*_3_-Cox, indicating that the CcoA-dependent high affinity (i.e., nM Cu) pathway including CcoA, is compromised even at growth-permissive low temperature. Supplementation of the growth medium with a redox active chemical (e.g., cysteine/cystine mixture) restores this high affinity Cu uptake pathway. On the other hand, addition of high concentrations (~ μM) of Cu^2+^ activates the low affinity (μM) CcoA-independent Cu uptake pathway, allowing Cu^2+^ incorporation into *cbb*_3_-Cox (Ekici et al., 2014; Figure 6B). Conceivably, besides CcoA, other component(s) (X) important for *cbb*_3_-Cox biogenesis may also be compromised in the absence of DsbA.

As summarized in Figure 6, this work showed that the respiratory chain of *R. capsulatus* becomes deficient in the absence of DsbA due to multiple defects, including decreased Ccm efficiency and impaired Cu incorporation into the active site of *cbb*_3_-Cox via the CcoA-dependent high affinity Cu uptake pathway. This biogenesis defect could be alleviated either by lowering DegP protease activity, or by providing chemical oxidants that restore the high affinity Cu uptake pathway, or by supplying high amounts of Cu^2+^ that activates a low affinity CcoA-independent Cu uptake pathway. Future studies will hopefully identify the *R. capsulatus* DsbA-dependent biogenesis component(s) that functions during the incorporation of Cu to *cbb*_3_-Cox.

## Author contributions

All authors have given approval to the final version of the manuscript. OO designed and performed experiments and analyzed data. AV designed and performed experiments, analyzed data and wrote the manuscript. BK-H and AP performed experiments. H-GK did critical revision of the manuscript. FD managed the project, supervised the study and wrote the manuscript.

### Conflict of interest statement

The authors declare that the research was conducted in the absence of any commercial or financial relationships that could be construed as a potential conflict of interest.

## Abbreviations

MPYE: Mineral-peptone-yeast extract enriched medium
MedA: Sistrom's minimal medium A
Res: Respiration
Ps: Photosynthesis
cyt *c*: cytochrome *c*
*cbb*_3_-Cox: *cbb*_3_-type cytochrome *c* oxidase
*bd*-Qox: *bd*-type quinol oxidase
NADI: a staining procedure for cytochrome *c* oxidase activity in colonies
GTA: gene transfer agent
Xgal: 5-bromo-4-chloro-3-indolyl-β-D-galactopyranoside
ONPG: *o*-nitrophenyl-β-galactoside
DBH_2_: decylbenzoquinol
PMFS: phenyl methyl sulfonyl fluoride
AEBSF: 4-(2-Aminoethyl) benzenesulfonyl fluoride hydrochloride.
